# Acupuncture Needle-Based Biosensors: Interface Engineering, Sensing Strategies, and Applications

**DOI:** 10.3390/bios16100582

**Published:** 2026-10-16

**Authors:** Yunjuan Wang, Yuhang Guo, Qing Liu, Yeli Xiao, Zhuo Xiang, Shunhong Xue, Xiaobing Kong, Nuo Shang, Zhifan Jiang, Jinjin Wang, Wenjing Xu

**Affiliations:** 1 School of Acupuncture-Moxibustion and Tuina, Beijing University of Chinese Medicine, Beijing 102401, China; 20250935355@bucm.edu.cn (Y.W.); 20240935327@bucm.edu.cn (Y.G.); 20240935328@bucm.edu.cn (Q.L.); xiaoyeli@bucm.edu.cn (Y.X.); 20260935330@bucm.edu.cn (Z.X.); 20230521028@bucm.edu.cn (S.X.); 2 School of Materials Science and Engineering, Peking University, Beijing 100091, China; 2201112400@stu.pku.edu.cn (X.K.); 2401112510@stu.pku.edu.cn (N.S.); 2301111461@stu.pku.edu.cn (Z.J.); 3 School of Physics and Laboratory of Zhongyuan Light, Zhengzhou University, Zhengzhou 450052, China; wjj3485815857@gs.zzu.edu.cn

**Keywords:** acupuncture needle-based biosensors, electrochemical sensing, surface-enhanced Raman spectroscopy, interface engineering, sensing strategies

## Abstract

Acupuncture needles, conventionally employed as therapeutic tools in Chinese medicine, have emerged as compelling substrates for constructing minimally invasive sensing platforms. Acupuncture needles’ unique attributes, including small size, sharp tip geometry, mechanical robustness, biocompatibility, and ease of insertion into tissue, render them attractive for in vivo, real-time monitoring applications. This review provides an overview of the recent advances in acupuncture needle-based biosensors (ANBSs), focusing on three interconnected aspects: interface engineering approaches, sensing strategies, and representative applications. We demonstrate electrochemical and surface-enhanced Raman spectroscopy (SERS)-based platforms as two principal classes of acupuncture needle-based biosensors, and survey their applications in neurotransmitter monitoring, disease biomarker detection, therapeutic drug monitoring, metabolite profiling, acupoint initiation investigation, as well as environmental contaminant screening and food safety assessment. Finally, we also discuss current challenges, including long-term instability, inadequate active sites, biofouling, signal drift, and insufficient reproducibility, while offering perspectives on future directions such as optimization of materials, miniaturization and multifunction, intelligent sensing strategies and clinical translation proposal. By bridging traditional acupuncture needles and advanced analytical science, this review not only provides a coherent framework for researchers in biosensors but also inspires the next generation of minimally invasive point-of-care diagnostic tools.

## 1. Introduction

Accurate and real-time monitoring of disease biomarkers, therapeutic drug concentrations, and key metabolites is of critical importance for early diagnosis, precision medicine, mechanistic elucidation of physiological processes, dynamic health surveillance, individualized therapeutic guidance, as well as food and environmental safety assessment. Over the past decades, biosensors, which integrate biological recognition elements with physicochemical transducers, have attracted extensive research interests owing to their capacity to deliver information directly from biological systems or specimens. Nevertheless, conventional biosensing devices still face several challenges, including invasive tissue damage, inadequate sensitivity and selectivity, and insufficient long-term stability within complex biological microenvironments, creating a trade-off between analytical performance and practical usability, which in turn poses a pressing demand for the exploration of novel substrates and innovative sensing interfaces to realize the next generation of minimally invasive and accurate biosensors.

Traditional Chinese acupuncture needles, as clinically mature and low-cost tools, have been widely applied in therapeutic interventions for thousands of years, attesting to their outstanding in vivo biocompatibility [1]. Moreover, these needles exhibit a range of superior structural and mechanical properties, including a small shaft diameter (typically 0.2–0.35 mm), a sharp conical tip, and excellent mechanical robustness, enabling minimally invasive and easy insertion into biological tissues, acting as an ideal platform for constructing percutaneous biosensors [2]. In addition, their metallic substrate and modifiable surface provide favorable conditions for interface functionalization and signal transduction, laying the foundation for the development of high-performance acupuncture needle-based biosensors (ANBSs). For example, compared with conventional glassy carbon electrodes, screen-printed electrodes, and other electrodes, acupuncture needles are better suited for constructing minimally invasive, in situ, and real-time sensing interfaces. Unlike carbon fiber microelectrodes, which are commonly used for in vivo measurements, acupuncture needles have a strong puncture ability and are not easily broken. Additionally, they are simple to manufacture and inexpensive compared to precious metal electrodes [3]. Therefore, by integrating acupuncture needles with functional sensing techniques, ANBSs represent compelling platforms for minimally invasive, highly sensitive and selective, in situ, and real-time monitoring of analytes.

**Figure 1:**
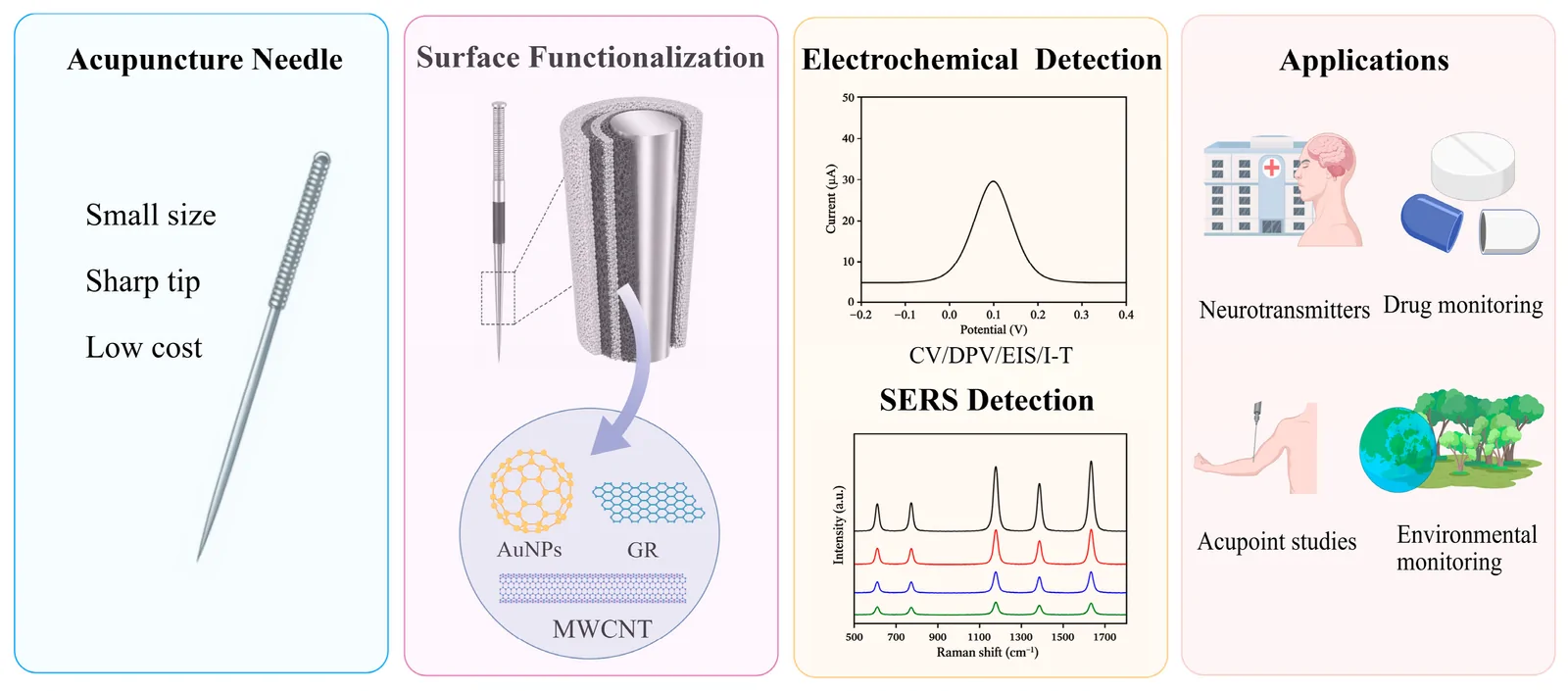
Schematic illustration of acupuncture needle-based biosensors, including interface engineering technologies, sensing strategies, and representative applications.

With the development of interface engineering technologies and analytical sensing strategies, ANBSs have gradually evolved into a distinctive and promising research field. By integrating functional nanomaterials (for example, metal nanomaterials, carbon nanomaterials, and conductive polymers), biorecognition elements (for example, enzymes, aptamers, and antibodies), antifouling interface design, and advanced signal processing strategies, researchers have modified the surface of conventional acupuncture needles to endow them with high sensitivity and selectivity. The overall design principles of ANBSs, including surface functionalization strategies, signal transduction modes, and representative application scenarios, are summarized in Figure 1. At present, electrochemical sensing and surface-enhanced Raman spectroscopy (SERS) sensing are two dominant sensing strategies. Electrochemical ANBSs offer advantages of low cost, rapid response, portability, and suitability for quantitative and, in some cases, real-time monitoring. SERS-based ANBSs can achieve highly sensitive molecular analysis through direct fingerprint detection, reporter-mediated sensing, or ratiometric strategies, with particular potential for multiplexed detection of both electroactive and non-electroactive targets. Benefiting from the above interface engineering technologies and sensing strategies, ANBSs have been applied to microvolume analysis, in situ sensing, and selected forms of in vivo monitoring. They have demonstrated remarkable versatility, enabling sensitive detection of a wide spectrum of analytes, including neurotransmitter such as dopamine [4,5,6,7] and serotonin [8,9]; physiological metabolite such as PH [10,11,12], rutin [13], and glucose [14,15,16]; clinically relevant protein biomarkers [17,18,19,20]; therapeutic drugs [3,5,21,22,23]; and environmental contaminants [24,25,26,27]. They have also demonstrated unique value in acupuncture mechanism research, enabling elucidation of local response to acupoint initiation and dynamic monitoring of signaling molecules in relevant brain regions [2,8,28]. These advancements demonstrate great application potential in cross-disciplinary fields of traditional Chinese medicine, biomedical engineering, analytical chemistry, and environmental detection.

Despite these rapid advances, the field of ANBSs still faces some unresolved challenges that hinder their further development and clinical translation, for example, long-term instability in complex biological microenvironments, insufficient active sites on needle surfaces, inevitable biofouling interference during in vivo monitoring, persistent sensing signal drift, and poor reproducibility, which restrict the performance optimization and practical applications of ANBSs [29,30,31]. Several previous reviews have examined microneedle biosensing, needle-type sensor technologies, and nanotechnology-enabled acupuncture from related but distinct perspectives. Ma et al. reviewed recent advances in microneedle-based biosensors, with emphasis on microneedle classification, fabrication methods, electrochemical and optical sensing strategies, and representative biomedical applications [32]. Vora et al. reviewed microneedle-based biosensing with particular attention to transdermal access to interstitial fluid, integration with wearable devices, real-time monitoring, and translational considerations [33]. Hu et al. further discussed microneedle sensors for point-of-care diagnostics and personalized medicine, highlighting electrochemical and optical “lab-on-a-microneedle” platforms and their potential for minimally invasive and continuous sensing [34]. From a broader perspective, Yang et al. analyzed two decades of needle-type sensor research using scientometric methods and identified three major research hotspots: multidisciplinary materials science, sensor miniaturization and integration, and biomedical engineering [35]. More recently, Liu et al. reviewed the integration of nanotechnology with traditional acupuncture, covering nano-enabled sensing, visualization and imaging, drug delivery, and therapeutic applications [1].

Although these previous reviews have provided useful perspectives, research specifically focusing on ANBSs remains relatively fragmented. In particular, the field has not yet been clearly organized around three core aspects: first, interface engineering approaches to tailor the needle surface morphology and composition for optimal sensing performance, anti-interference, and biocompatibility; second, sensing strategies to generate and readout signals and, thus, determine the target capture efficiency, signal-to-noise ratio, and selectivity of ANBSs in complex biological microenvironments; and third, application-oriented performance validation across diverse use cases.

To address these challenges, this review provides an overview of the latest research progress of ANBSs. We firstly summarize the interface engineering technologies and mainstream sensing strategies for high-performance ANBSs, and then classify and elaborate on their representative applications in biomedical detection, acupoint mechanism research, food safety, and environmental pollution assessment. Meanwhile, this review thoroughly analyzes the key challenges, limiting the practical application of ANBSs from the perspectives of material properties, structural design, and interference. Moreover, the future directions, including advanced material optimization, surface/interface engineering, and intelligent sensing strategies are discussed. By bridging traditional acupuncture tools with modern advanced analytical sensing technologies, this work aims to establish a structured framework for ANBS research, provide valuable references for biosensor researchers in related fields, and inspire the development of new-generation minimally invasive monitoring tools.

### 1.1. Literature Search Strategy and Review Scope

Relevant publications were identified through searches of PubMed and Web of Science, with the final search updated in August 2026. Relevant publications were identified using the search formula “TS = ((‘acupuncture needle’ OR ‘acupuncture needle electrode’) AND (‘sensor’ OR ‘SERS’ OR ‘surface-enhanced Raman spectroscopy’))”. The core literature search focused primarily on studies published from 2015 to 2026. Reference lists of relevant primary studies and review articles were also examined to identify additional publications related to acupuncture needle-based sensing.

Primary studies were considered for inclusion when a traditional acupuncture needle or an acupuncture-needle-derived structure served as the sensing substrate, working electrode, probe carrier, or an integrated component of the sensing system, and when analytical performance or application validation was reported. Studies based solely on conventional syringe needles, wire microelectrodes, or patch-type microneedle arrays without an acupuncture-needle component were not considered ANBS studies and were used only for contextual comparison when relevant.

### 1.2. Definition and Classification of ANBSs

The terminology used for needle-based biosensors was not always consistent in the literature. Terms such as “needle”, “microneedle”, and “smart needle” might refer to different device structures. Therefore, in this review, ANBSs were mainly defined according to whether an acupuncture needle was used as the sensing component of the device.

A device was considered an ANBS when an acupuncture needle, or a structure directly developed from an acupuncture needle, was incorporated into the sensing system and played a direct role in signal generation or analyte detection. For example, the acupuncture needle might serve as the working electrode, the sensing substrate of a FET-based device, or the carrier of functional materials for optical or SERS detection. Additional components might also be included for signal measurement, protection, or device integration, as long as the acupuncture needle remained the main sensing component.

In contrast, conventional syringe needles, wire microelectrodes, and microfabricated microneedle patches were not included as ANBSs when an acupuncture needle was not used as the main sensing substrate. The term “microneedle” was particularly important because some studies used it to describe functionalized acupuncture needles, whereas many conventional microneedle biosensors were based on separately fabricated microscale needles or arrays. Therefore, the devices discussed in this review were classified according to their actual structure and the role of the acupuncture needle rather than only according to the terminology used in the original studies. The classification used in this review is summarized in Table 1.

## 2. Sensing Strategies and Interface Engineering of Acupuncture Needle-Based Biosensors

The transformation of a conventional acupuncture needle into a functional biosensing platform critically depended on sensing strategies and interface engineering approaches. These two aspects were inherently intertwined, as the selected sensing strategy dictated the required interfacial properties, while the interface engineering approaches, encompassing material selection, structural design, and assembly governed the achievable sensitivity, selectivity, and stability of the resulting ANBSs. In this section, we provide an overview of the two predominant sensing paradigms, namely electrochemical and optical (surface-enhanced Raman scattering) biosensing, and subsequently discuss the corresponding interface engineering technologies tailored to each strategy.

### 2.1. Electrochemical ANBSs: Sensing Strategies and Interface Engineering

#### 2.1.1. Electrochemical Sensing Principles and Device Designs of ANBSs

Electrochemical sensing is one of the most widely used strategies in ANBS research, offering operational simplicity, high sensitivity, low cost, and minimal invasiveness suitable for in vivo applications. Conventional electrochemical ANBSs are configured as a three-electrode system comprising a working electrode (WE), a reference electrode (RE), and a counter electrode (CE). In this configuration, the stainless-steel acupuncture needle serves as the working electrode. In order to achieve desirable sensing performance, the acupuncture needles typically necessitate rational structural design and modification. The resulting functionalized acupuncture needles work as the sensing interface where target recognition and electrochemical reactions take place, thereby eliminating the need for additional invasive probes and facilitating direct insertion into the tissue of interest. The reference electrode (commonly a saturated calomel electrode or Ag/AgCl) provides a stable and known potential, while the counter electrode (typically platinum, gold, or graphite) completes the electrical circuit of ANBSs [37].

**Figure 2:**
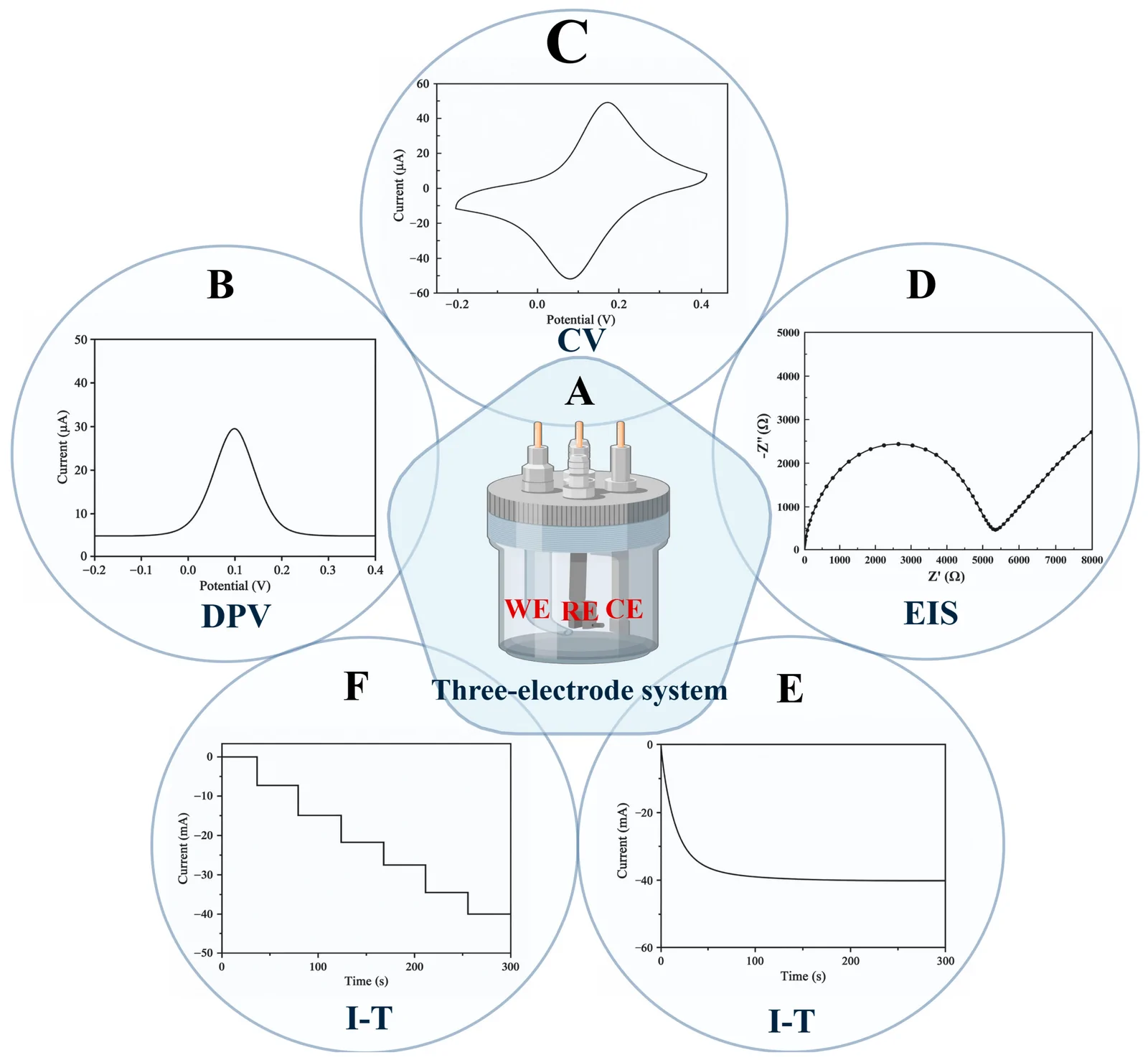
Representative three-electrode configuration and electrochemical readout methods used in electrode-based ANBSs. (**A**) Conventional three-electrode system comprising a working electrode (WE), reference electrode (RE), and counter electrode (CE). (**B**) DPV; (**C**) CV; (**D**) EIS; and (**E**,**F**) chronoamperometric responses. These examples show commonly used electrochemical readout methods and do not represent all ANBS device configurations.

The electrochemical performance of ANBSs is characterized using complementary techniques. Cyclic voltammetry (CV) and electrochemical impedance spectroscopy (EIS) are employed to assess interfacial modification efficacy. Therein, CV evaluates electron-transfer kinetics and conductivity enhancement, while EIS provides charge-transfer resistance and diffusion-related information [16]. For quantitative analysis and dynamic monitoring, differential pulse voltammetry (DPV) offers high sensitivity for trace analyte detection, and chronoamperometry (I-t) enables real-time current recording for continuous monitoring [38]. Representative studies, such as AuNPs/PEDOT-modified ANs for reduced nicotinamide adenine dinucleotide (NADH) sensing [39], illustrate this complementary use: CV is used to compare the redox responses of the bare and modified electrodes; DPV is used for quantitative analysis; and EIS confirms the decrease in charge-transfer resistance after modification. Representative electrochemical methods used in conventional ANBSs are shown in Figure 2.

In addition to conventional electrochemical sensing, FET-based ANBSs represent a distinct electrical sensing approach and do not operate within the conventional three-electrode framework. In FET-based systems, changes at the sensing interface modulate the electrical characteristics of the transistor channel rather than being directly measured as faradaic current, electrode potential, or impedance. Zhou et al. developed an acupuncture needle-based FET neuroprobe in which the needle was modified with insulating and gold layers, while reduced graphene oxide served as the sensing channel. An aptamer was further introduced for selective neurotransmitter detection [6]. The device retained a needle-like structure and enabled in vivo sensing, although an external Ag/AgCl reference electrode was still required during measurement. A dual-needle FET configuration was later developed for pH monitoring. In this system, one needle contained the CNT-based FET structure, while a separate polyaniline-modified Pt gate acted as the pH-sensitive component [12]. The sensor showed a response of 53.7 mV/pH over the pH range of 4.0–9.0 and was used for real-time monitoring of pH changes in the rat brain.

Other integrated designs remained electrochemical in their signal-transduction principles. In a needle-in-needle sensor for etoposide monitoring, a functionalized acupuncture needle was placed inside a syringe needle [22]. The inner acupuncture needle acted as the working electrode, an Ag/AgCl wire served as the reference electrode, and the outer syringe needle functioned as the counter electrode. Therefore, although its physical architecture differed from that of a conventional electrode assembly, the needle-in-needle system retained a three-electrode electrochemical configuration. The outer syringe needle served as a protective sheath during tissue insertion. In another study, a K^+^ sensor combined a K^+^-selective membrane/rGO-modified acupuncture needle with a spiral Ag/AgCl reference electrode inside a glass capillary [36]. The acupuncture needle functioned as the working electrode, while the spiral Ag/AgCl wire acted as the integrated reference electrode. This configuration integrated the working and reference electrodes within a compact structure and was used for small-volume sample detection.

Overall, the reported ANBS configurations differed not only in their physical architectures but also in their signal-transduction mechanisms. Conventional voltammetric/amperometric systems and the needle-in-needle platform retained three-electrode electrochemical operation, whereas the capillary-integrated K^+^ sensor employed a potentiometric working/reference-electrode arrangement. By contrast, single- and dual-needle FET platforms relied on transistor-based electrical transduction rather than conventional electrochemical readout.

#### 2.1.2. Modification Material and Functionalization Strategies of ANBSs

**Figure 3:**
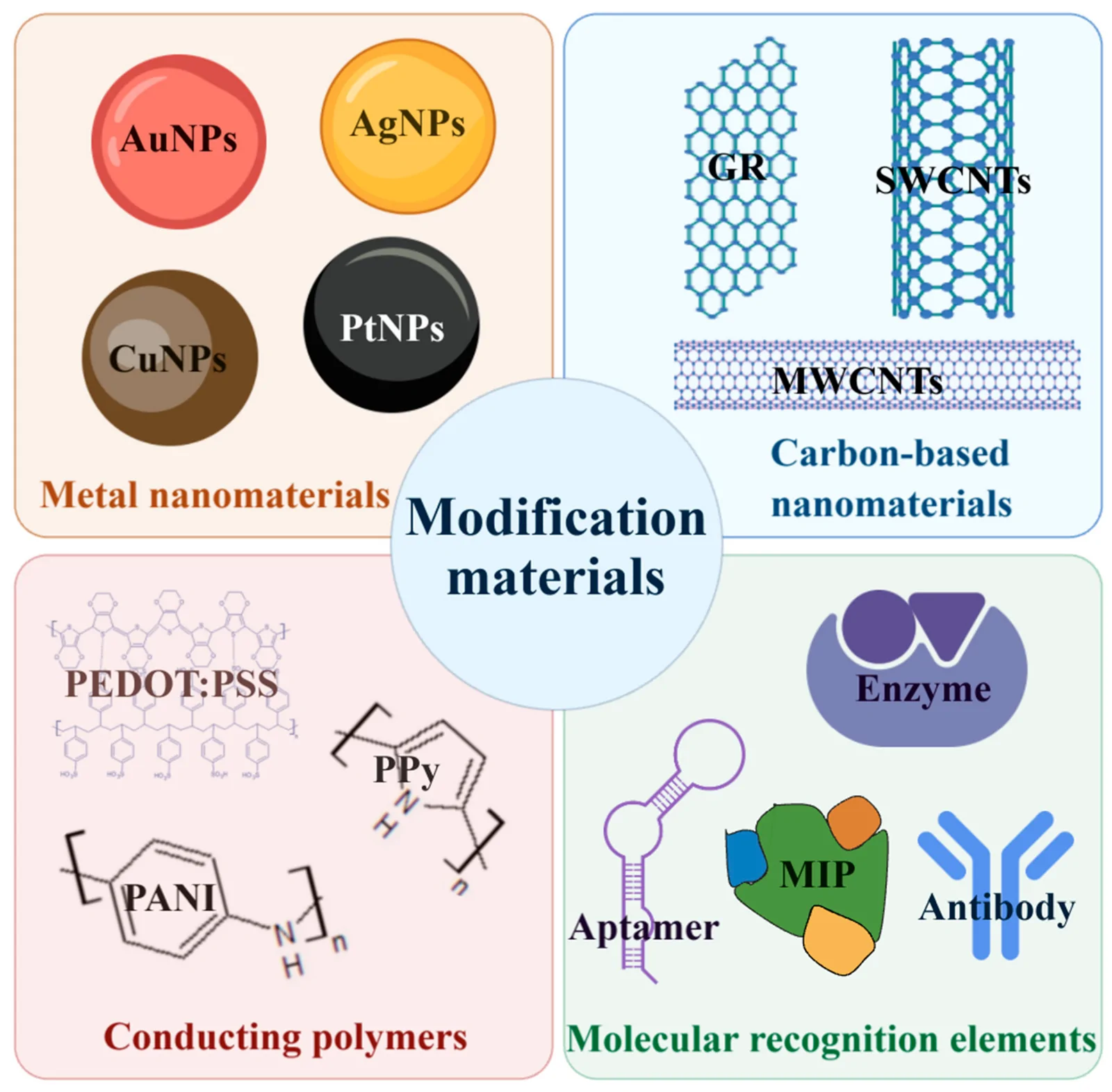
Classification of modification materials for acupuncture needle-based biosensors.

To realize sensitive and selective detection on electrochemical ANBSs, multilayered functional interfaces on the acupuncture needle-based working electrode were constructed. The resulting functionalized acupuncture needles typically exhibited an integrated bilayer architecture: a recognition layer that incorporated biorecognition elements (for example, enzymes, antibodies, and aptamers) or molecularly imprinted polymers (MIPs) to confer specific recognition toward the target analyte; a transduction layer, composed of conductive materials such as metals, carbon nanomaterials, or conducting polymers, which enhanced conductivity, enlarged the electroactive surface area, accelerated electron transfer, and endowed catalytic activity. The representative functional materials used for constructing electrochemical ANBSs are summarized in Figure 3.

The recognition layer plays a direct and decisive role in determining specificity of ANBS performance. Antibodies, enzymes, aptamers, and molecularly imprinted polymers (MIPs) are the most commonly employed biorecognition elements, owing to their high specificity in recognizing target analytes or their specific catalytic activity toward target molecules. Antibodies are widely used for protein and pathogen detection owing to their high affinity and selectivity. For example, anti-SARS-CoV-2 coronavirus nucleocapsid protein (N protein) monoclonal antibodies were immobilized on AuNP/poly(EDOT-COOH-co-EDOT-N3)-modified acupuncture needles through EDC/NHS chemistry, while PEGylation was introduced to improve antifouling capability, enabling ultrasensitive detection of SARS-CoV-2 antigens [40]. Enzymes provided both substrate-specific recognition and catalytic signal conversion. For example, a laccase-modified acupuncture needle sensor was developed for monitoring brain tissue oxygen partial pressure [41]. Aptamers, owing to their broad target adaptability and facile functionalization, had also emerged as recognition elements for AN-based microelectrode platforms. Additionally, aptamers had been employed both in field effect transistor neuroprobes for real-time in vivo detection of dopamine and neuropeptide Y [6]. Moreover, aptamers could be integrated with gold nanoparticles (AuNPs) and metal–organic framework (MOF) materials to construct self-calibrating needle-electrode sensors for the simultaneous detection of Cd(II) and Vibrio parahaemolyticus [26]. Among these recognition strategies, molecularly imprinted polymers (MIPs) are particularly representative in ANBSs because of their facile preparation, high stability, relatively low cost, and high selectivity. MIPs are synthetic recognition materials prepared by polymerizing functional monomers around a target molecule. After the template is removed, specific recognition cavities remain in the polymer layer. These cavities could recognize the target according to its size, shape, and chemical properties. When the target is bound again, changes in the electrochemical interface could be detected and used for quantitative analysis. However, their sensing performance is affected by factors such as monomer selection, template removal, accessibility of the recognition sites, nonspecific adsorption, and reproducibility of the imprinted layer [42]. MIPs had been widely applied to the detection of small-molecule drugs, neurotransmitters, and protein biomarkers, including promethazine [21], chlorpromazine [3], dopamine [7], mesothelin [17], β-amyloid42 [18], and uric acid [43]. Boronic acid derivatives could also participate in synthetic recognition, either as functional monomers in molecular imprinting or through reversible interactions with cis-diol-containing molecules [44]. For example, 4-mercaptophenylboronic acid was used to assist the immobilization of the SARS-CoV-2 spike protein during the preparation of a surface-imprinted acupuncture-needle sensor [20]. Therefore, boronic acid-based chemistry could be used as a complementary strategy in the construction of recognition interfaces, although its role depends on the target molecule and sensing design. In addition to antibodies, enzymes, aptamers, and MIPs, the specific catalytic reactions of nanomaterials could be employed to generate catalytic signals for reflecting the recognition of target analytes.

As the indispensable interfacial component bridging the biorecognition element and the underlying electrode, the transduction layer governs the efficient conversion of biochemical binding events into quantifiable electrochemical readouts, thereby critically determining the overall sensitivity, detection limit, and operational reliability of ANBSs. Metallic nanomaterials, particularly gold nanoparticles (AuNPs), platinum nanoparticles (PtNPs), and their bimetallic or metal oxide composite structures, are extensively employed for constructing transduction interfaces of ANBSs. For example, AuNPs are favored for their high stability, good catalytic activity, and superior conductivity [3,45,46]. Tang et al. electrodeposited AuNPs onto the tip of an acupuncture needle, followed by cyclic voltammetry reduction of graphene oxide to obtain a graphene-modified needle (G-AN) [4]. This nanomaterial functionalized platform enabled sensitive detection of dopamine (DA) with a limit of detection (LOD) of 0.24 μM and successfully achieved DA measurement in human serum, laying a foundation for exploring molecular events in acupuncture medicine. To further enhance catalytic performance, bimetallic interfaces had been developed. For instance, Xiao et al. sequentially electrodeposited Au and Pt nanoparticles onto acupuncture needles [16]. The resulting Pt/Au/AN electrode exhibited a cabbage-like morphology and superior electrocatalytic activity toward glucose oxidation compared with monometallic electrodes, achieving a wide linear range of 0.1–35 mmol·L^−1^ and an LOD of 0.0763 mmol·L^−1^, demonstrating the synergistic effect of bimetallic contacts. To improve selectivity in complex matrices, bimetallic interfaces had been integrated with MIPs. Chen et al. electrodeposited Au/Cu bimetallic nanoclusters onto an acupuncture needle and subsequently constructed an MIP film using 3-aminophenylboronic acid as the functional monomer [3]. In this design, the Au/Cu nanoclusters enhanced electrocatalytic performance, whereas the MIP layer provided selective recognition toward chlorpromazine. The resulting sensor exhibited two linear ranges (0.1–100 μM and 100–1000 μM) and an LOD of 0.07 μM in human serum, illustrating the effectiveness of combining catalytic interfaces with artificial recognition layers.

Carbon-based nanomaterials are also a fundamental transducer layer of ANBSs. Graphene, reduced graphene oxide (rGO), and multiwalled carbon nanotubes (MWCNTs) provide large specific surface areas, excellent conductivity, and favorable biocompatibility, thereby promoting electron transfer [47]. For example, CNTs could form porous surface networks on the needle tip and thus increase the number of available reaction sites. However, when used alone, CNTs might exhibit poor adhesion to the metallic needle surface and are prone to peeling off during tissue insertion. To address this limitation, they are often combined with conducting polymers such as poly(3,4 ethylenedioxythiophene) (PEDOT) to balance conductivity and mechanical robustness. Li et al. developed a microsensor by modifying CNTs onto the needle tip using PEDOT as a conductive “glue” to prevent CNT detachment [8]. The PEDOT/CNT/AN sensor was directly inserted into rat bodies for real-time monitoring of serotonin (5-HT) at the Zusanli (ST36) acupoint, achieving LODs of 50 nM in PBS and 78 nM in cell medium. This work reported for the first time that the functional acupuncture needle could be directly probed into rat bodies for real-time monitoring of the signal molecules in acupoints. To achieve simultaneous detection of multiple analytes, Zhan et al. constructed a MWCNT/PDA/AuNP/AN sensor by sequentially electrodepositing AuNPs, electropolymerizing dopamine (PDA), and finally depositing carboxylated MWCNTs [5]. In this architecture, AuNPs and MWCNTs improved conductivity and electrocatalytic performance, whereas PDA enhanced interfacial adhesion and structural stability. The resulting sensor enabled simultaneous detection of dopamine, acetaminophen, and chlorpromazine in human serum and urine, with LODs of 0.015, 0.012, and 0.022 μM, respectively.

In addition to metals and carbon nanomaterials, conducting polymers played multiple roles in ANBSs, serving not only to improve conductivity but also to stabilize surface coatings and promote interfacial electron transfer. PEDOT and its derivatives were primarily used as conductive and stabilizing interlayers. As described above, PEDOT acted as a conductive “glue” to immobilize CNTs on the needle tip [8]. Later studies further confirmed its interfacial advantages. For example, in a Cd(II) sensor, PEDOT:PSS was electrodeposited as a conductive polymer film to enhance electron transfer capability and conductive efficiency [25]. In an NADH sensor, the AuNP/PEDOT-modified needle exhibited stronger electrochemical responses than the bare needle or one component interfaces, indicating that PEDOT could effectively cooperate with metallic nanostructures to accelerate electron transfer [39]. Additionally, polyaniline (PANI) had been widely considered as a candidate for pH sensing owing to its excellent pH-dependent reversible charge transfer properties. A MoS_2_/PANI-functionalized acupuncture needle sensor was developed for in vivo monitoring of pH changes in rat brain cerebrospinal fluid. The PAN/MoS_2_/AN electrode exhibited a near-Nernstian response with a sensitivity of 51.2 mV/pH over a broad pH range of 3.0–9.0 [10]. In another study, a pH microneedle modified with platinum black, AuNPs, and PANI achieved an even higher response slope of −57.4 mV/pH over pH 4.0–9.0 [11]. Different from PEDOT and PANI, polydopamine (PDA) was more commonly utilized as a universal adhesive and multifunctional surface modification layer. In the AuNP/PDA/AN sensor, PDA strengthened the adhesion and stability of gold nanoparticles on the needle surface and facilitated subsequent functionalization [46].

Therefore, the synergy between the recognition and transducer layer converted the traditional needle into a highly sensitive and selective electrochemical electrode capable of real-time quantitative monitoring.

### 2.2. Optical ANBSs: Sensing Strategies and Interface Engineering

Although electrochemical detection remains predominant, acupuncture needles possess intrinsic advantages for optical sensing platforms. Their slender geometry, mechanical robustness, sharp conical tip, and ease of tissue insertion make them attractive miniaturized substrates for surface-enhanced Raman spectroscopy (SERS), a technique that amplifies Raman scattering signals through localized surface plasmon resonance excited on nanostructured noble metal surfaces. The functionalization of the needle surface with plasmonic nanostructures, typically gold or silver nanoparticles, nanoislands, or nanostars, generates intense electromagnetic fields in nanoscale gaps (“hot spots”) upon laser excitation. When target molecules are located near these hot spots, their Raman signals could be strongly enhanced. Depending on the sensing design, SERS-based ANBSs might operate through direct molecular fingerprint detection or through indirect reporter-mediated or ratiometric strategies. Therefore, both electroactive and non-electroactive analytes could be detected without relying on electrochemical redox activity. Furthermore, the high spectral resolution of Raman spectroscopy allows different characteristic bands or Raman reporters to be distinguished within a single measurement, providing potential for multiplexed analysis.

Furthermore, the acupuncture needle configuration provides advantages for SERS-based sensing. The small needle tip is suitable for localized sampling from small-volume biological specimens and could also be inserted into tissues or biological fluids to interact with target molecules at defined locations. Depending on the optical configuration, Raman spectra might be acquired either directly at the sensing site or after withdrawal of the needle. Therefore, SERS-based ANBSs provide a minimally invasive approach for spatially resolved molecular analysis. However, continuous real-time Raman acquisition in living tissue had not yet been demonstrated for most reported platforms.

A representative early study, reported in 2017, demonstrated an acupuncture needle-based surface-enhanced resonance Raman spectroscopy (SERRS) platform for dopamine detection [48]. In that work, the needle surface was decorated with Au nanoparticles and further functionalized with a citrate-Fe(III) structure, which simultaneously acted as a molecular capture unit and a resonance-amplifying component through formation of a CA-Fe(III)-DA complex. By combining plasmonic enhancement with resonance Raman amplification, this design improved the intrinsically weak Raman response of dopamine and enabled sensitive detection in biological samples, achieving detection limits of 0.1 nM in serum and 2.5 nM in cerebrospinal fluid. However, this work primarily demonstrated SERRS analysis in biological samples rather than continuous in vivo Raman monitoring.

To improve SERS sensitivity and reproducibility, a study in 2018 developed an amphiphilic functionalized Ag acupuncture needle by decorating the Ag needle with polyvinylpyrrolidone (PVP)-stabilized Au nanoparticles [49]. In this configuration, the PVP coating not only facilitated the formation of a highly uniform Au nanoparticle array on the surface of the Ag needle via its adhesiveness and steric properties but also promoted the adsorption of the target analytes in both the oil and aqueous phases owing to its amphiphilic nature. The resulting AuNP-Ag needle system (Au-AgN) exhibited pronounced SERS enhancement arising from multiple plasmonic couplings, including from interparticle and particle−film interactions. Notably, these Au-AgN ANBSs could be directly inserted into the multiphase system for in situ laser detection, and SERS measurements at different spatial positions on the same Au-AgN needle allowed simultaneous monitoring of targets analytes in the distinct phase, thereby minimizing sample disturbance and providing more accurate multiphase information.

Building on this concept, a 2019 study on malachite green introduced a microstructure-confined design to improve insertion stability [50]. A SERS-active Ag acupuncture needle was engraved with arrays of micron-sized grooves that trapped polyvinylpyrrolidone (PVP)-stabilized Au nanoparticles. These microgrooves reduced the loss of AuNPs during tissue insertion and also facilitated optical localization under the Raman microscope. In addition, PVP associated with the AuNPs served as an inherent internal calibration standard, thereby reducing fluctuations in absolute SERS intensity. The platform achieved a detection limit of 0.1 nM for malachite green. Importantly, Raman spectra were collected after the needle was withdrawn from the fish tissue; therefore, this platform was more accurately described as in vivo sampling followed by ex vivo SERS readout rather than continuous real-time in vivo monitoring.

In another study, Au nanoparticles were assembled on the needle surface to generate abundant SERS hot spots while avoiding nanoparticle aggregation, resulting in a reproducible SERS response. The strong interaction between ATP and macrocyclic amine (MA), driven by electrostatic adsorption, hydrogen bonding, and π-π stacking, enabled the functionalized needle to serve as a SERS platform for ATP detection at 25 nM [51]. Unlike direct molecular-fingerprint SERS, this platform represented an indirect reporter-mediated sensing strategy.

Recent advances in acupuncture needle-based SERS platforms had further shifted from simple plasmonic nanoparticle decoration toward integrated probe engineering and multiparameter sensing in biological tissues. In 2021, Chen et al. developed a SERS chips-in-acupuncture needle platform by embedding microscale magnetic SERS probes into a magnetic pit constructed inside the acupuncture needle [52]. Unlike conventional surface-modified SERS needles, this internal integration strategy effectively protected the SERS probes from mechanical loss during tissue insertion. The magnetic pit enabled stable anchoring of multiple SERS probes, allowing simultaneous assessment of several physiological parameters, including pH, redox potential, and nitric oxide (NO), through in vivo interaction followed by ex vivo Raman readout.

More recently, Xiong et al. reported a SERS-active acupuncture needle for simultaneous detection of hydrogen peroxide (H_2_O_2_) and redox potential in acute wounds under different sugar intake conditions [53]. In this work, two microgrooves were fabricated near the needle tip through electrochemical corrosion, providing confined sites for integrating gold nanoshell-based SERS probes. A 4-mercaptophenylboronic ester (4-MPBE)-based probe was employed for H_2_O_2_ sensing, while a redox-responsive Raman probe was introduced for redox potential assessment. The groove-confined architecture improved probe immobilization stability during tissue penetration and enabled minimally invasive interaction with the wound microenvironment in vivo, followed by ex vivo SERS readout.

Following this strategy, Wang et al. further developed a SERS-active microneedle for simultaneous detection of oxidative capacity (OC) and redox potential (RP) in rats with high glucose-induced stress [54]. Two independent SERS probe systems were integrated into separate grooves on an acupuncture needle, where gold nanoshells served as plasmonic substrates. The p-phenylenediamine (p-PDA)-based probe responded to oxidative capacity, whereas an anthraquinone derivative was used for redox potential sensing. The probes responded to the tissue environment in vivo, whereas the SERS spectra were collected after withdrawal of the microneedle.

In addition to redox-related sensing, Zhong et al. recently reported a Au nanoparticle-functionalized acupuncture needle SERS platform for ratiometric detection of pH and nitroreductase (NTR) in solution and intact tissue samples [55]. The acupuncture needles were modified with gold nanoparticles and further functionalized with 5-mercapto-2-nitrobenzoic acid (MNBA), in which the carboxyl group and nitro group served as pH- and NTR-responsive groups, respectively. By employing the Au-S bond signal as an internal reference, the proposed ANs@Au NPs@MNBA platform achieved quantitative dual-parameter analysis in both the solution and intact tissue samples without destructive sample pretreatment.

Quantitative SERS measurements could be affected by the distribution of plasmonic nanostructures, measurement position, and sample matrix. These factors might contribute to signal variation between different measurement sites or different sensors. Microgroove structures, internal reference signals, and ratiometric strategies had been introduced to improve signal reproducibility and quantitative analysis. In addition, measurements in complex biological samples might be affected by matrix-related interference compared with those obtained in simple solutions [49,51,55].

Overall, current optical ANBSs are predominantly based on SERS or SERRS. Plasmonic Au/Ag nanostructures are employed to generate electromagnetic hot spots; recognition elements or Raman-responsive probes are incorporated according to the sensing strategy; and interfacial or structural engineering is further implemented to improve coating stability, measurement reproducibility, and quantitative performance. However, most reported SERS-based ANBSs still rely on in vivo interactions or sampling followed by ex vivo Raman readout.

### 2.3. Comparative Analysis of Electrochemical and Optical ANBSs

#### 2.3.1. Difference Between Electrochemical and Optical ANBSs

Electrochemical and SERS-based ANBSs operate on fundamentally distinct signal transduction principles. Electrochemical systems convert interfacial recognition or catalytic events into current, potential, conductance, or impedance signals, whereas optical systems translate target binding into changes in Raman intensity, plasmonic response, or other optical outputs [56,57]. This distinction determines their respective device architectures, analytical performance, and application scenarios.

From a practical implementation perspective, electrochemical sensing currently represents the more mature and widely adopted route, primarily because the metallic acupuncture needle could serve directly as the working electrode, thereby simplifying device fabrication, electrical connection, and system miniaturization. Electrochemical systems are also generally more cost-effective, portable, and minimally invasive. In contrast, optical platform typically requires more sophisticated instrumentation, rigorous signal calibration, and spectral interpretation, which could limit their routine deployment. Nevertheless, SERS-based ANBSs offer several advantages for optical sensing, including high sensitivity, characteristic Raman signals, and the ability to detect both electroactive and non-electroactive analytes. The narrow Raman bands could facilitate signal discrimination and multiplexed analysis, while the weak Raman scattering of water is favorable for measurements in aqueous biological environments [48,51]. However, not all SERS-based ANBSs are label-free because some platforms rely on Raman reporters or responsive probes.

#### 2.3.2. Similarity of Electrochemical and Optical ANBSs

Despite the forementioned differences, the analytical performance of both electrochemical and optical ANBSs is critically governed by interfacial materials and micro/nanostructural design. Among various modification strategies, the incorporation of noble metals, particularly gold (Au) and silver (Ag), represents one of the most widely adopted approaches in both electrochemical and optical ANBSs. These noble metal nanostructures serve to enlarge the electroactive area, accelerate charge transfer, amplify local optical fields, and provide selective recognition sites [56].

Structural design plays an equally important role in determining sensor performance. Micro- and nanostructural engineering could increase the effective surface area of the needle tip, enhance the loading of functional materials, or improve the stability of the sensing interface. For electrochemical ANBSs, this translates into more active sites and more efficient electron transfer. For example, Jia et al. grew Ni_6_MnO_8_ nanoflakes directly on an acupuncture needle, creating a highly porous three-dimensional network that afforded a large electroactive surface area and abundant catalytic sites, yielding an ascorbic acid sensor with a high sensitivity of 3106 μA·mM^−1^·cm^−2^ [58]. For optical ANBSs, nanostructured plasmonic surfaces intensified light–matter interactions, while specific structural designs could also improve the stability of plasmonic materials on the needle surface. Zhou et al. used focused ion beam milling to create precisely defined microgrooves on a silver acupuncture needle, followed by assembly of AuNPs inside these grooves [50]. This design confined AuNPs within the microgrooves, improving nanoparticle retention during tissue insertion and providing more reproducible SERS-active regions. Thirty spectra collected randomly from the groove regions showed an RSD below 10% for the major Raman band, indicating good spot-to-spot reproducibility.

#### 2.3.3. Performance Evaluation and Optimization of ANBSs

The analytical performance of reported ANBSs varies because of differences in target analytes, sensing methods, sample matrices, and validation conditions. Therefore, numerical values such as the limit of detection (LOD) should not be compared without considering the experimental conditions. To provide a clearer comparison, the main analytical characteristics of electrochemical and SERS-based ANBSs are summarized separately in Table 2 and Table 3.

For each study, the main information on sensor design, target analyte, sample or test condition, linear range, LOD, performance evaluation, and validation level is summarized when available. Parameters that were not clearly reported in the original studies are marked as “NR” (not reported). Because the included studies involve different analytes and sample types, the original concentration units are retained.

The validation conditions also differed considerably among studies. Some sensors were tested only in standard solutions or spiked biological samples, whereas others were evaluated in real biological specimens, ex vivo tissues, or living animals. These different validation settings should not be considered equivalent. In particular, good performance in buffer or spiked samples did not necessarily indicate the same performance during in vivo sensing. For example, the needle-in-needle etoposide sensor showed different calibration responses in buffer and rat peritoneal fluid, indicating that sensor performance could be affected by the sample matrix and should be interpreted together with the validation conditions [22].

Overall, the reported studies showed that a low LOD alone did not fully reflect the practical performance of an ANBS. Some sensors achieved very high sensitivity in buffer or spiked samples, whereas fewer studies were evaluated in living animals. Moreover, response time, long-term drift, recovery after fouling, and repeatability after implantation were inconsistently reported. Therefore, the analytical performance of ANBSs should be interpreted together with the sample matrix, validation setting, and stability of the sensing system. Beyond analytical sensitivity and validation conditions, the practical performance of ANBSs also depended on the stability of the sensing interface during tissue insertion and operation. Accordingly, structural protection, insulation, antifouling modification, and other stabilization strategies had been introduced to improve the reliability of ANBSs under practical sensing conditions.

Mechanical robustness is an important consideration for ANBSs because functionalized needles must penetrate tissue while maintaining both the structural integrity of the needle and the stability of the sensing interface. For example, Vieira et al. evaluated porous acupuncture needles using compression testing and a tissue-mimicking ballistic gel [41]. The modified needles showed no obvious bending or fracture during penetration, indicating that surface modification did not necessarily compromise the mechanical integrity of the needle. However, quantitative evaluation of coating loss during tissue insertion remained limited in most studies.

To address this challenge, researchers had developed various stabilization strategies. For example, Liu et al. proposed a “needle-in-needle” configuration, in which a functionalized acupuncture needle serving as the working electrode was inserted into a larger hollow outer needle [22]. During tissue penetration, the outer needle protected the sensing interface from mechanical abrasion, enabling continuous in vivo monitoring of etoposide in the rat peritoneal cavity with excellent stability. Similarly, Zhou et al. introduced microgrooves on a silver acupuncture needle to retain Au nanoparticles and reduce their loss during tissue insertion [50]. These examples show that structural protection could improve the stability of functional coatings during insertion.

Insulation and encapsulation are also critical for optimizing the performance of ANBSs, as they could not only preserve the stability of the sensing interface during operation but also suppress background interference from complex biological environments. In most reported designs, insulating materials such as epoxy resin are applied to the needle body, leaving only a short segment near the tip exposed as the active sensing region. This partial insulation spatially confines the sensing area, reduces background interference from the needle shaft, and improves measurement precision and reproducibility. However, the stability of these insulating layers after repeated tissue insertion have rarely been evaluated in detail.

In addition to physical encapsulation, chemical insulation and antifouling protection are equally important for preserving sensor performance in complex biological matrices. Semipermeable or antifouling polymer coatings, such as poly(ethylene glycol) (PEG)-based films, could be introduced onto the sensing interface to suppress nonspecific adsorption and reduce interference. PEG forms a dense hydration layer through hydrogen bonding with water molecules, creating a barrier against protein adsorption and other biological contaminants. Chen et al. incorporated a PEG-based antifouling layer into a dual-functional acupuncture needle biosensor for SARS-CoV-2 antigen detection; the PEG-modified surface reduced nonspecific protein adsorption by 82% relative to unmodified controls, and the sensor maintained 89.5% of its initial current response after 1000 cycles in serum [40]. These results demonstrated the potential of antifouling modification for improving sensor stability in complex biological samples, although this platform was not evaluated during prolonged in vivo implantation.

Operational stability should also be considered separately from storage stability. For example, the needle-in-needle etoposide sensor retained 93.6% of its initial response after 120 min of measurements in rat peritoneal fluid [22]. In another study, a dual-needle FET sensor showed a similar pH response before and after 1 h implantation in the rat brain [12]. Such pre- and post-insertion measurements provided useful information on whether the sensing interface remained functional after exposure to biological tissues.

Several issues still require further investigation. Repeated tissue insertion might cause coating delamination or material shedding, but the amount of released nanomaterials is rarely measured directly. Therefore, possible tissue exposure to detached Au, Ag, CNTs, or other functional materials should be regarded as a potential biosafety concern. In addition, the effects of the local tissue response after repeated or prolonged implantation remain insufficiently studied. Future work should pay greater attention to coating retention, signal drift and long-term stability, and tissue response during in vivo use.

Overall, the sensing performance of ANBSs is determined by the interplay among needle configuration, interface engineering, signal-transduction mode, structural stability, and antifouling capability. Current studies have demonstrated several useful strategies, including protective needle structures, microgroove confinement, insulation, and antifouling coatings. However, further evaluation of mechanical stability, coating retention, and long-term in vivo performance is still needed for the practical development of ANBSs.

## 3. Applications of ANBSs

**Figure 4:**
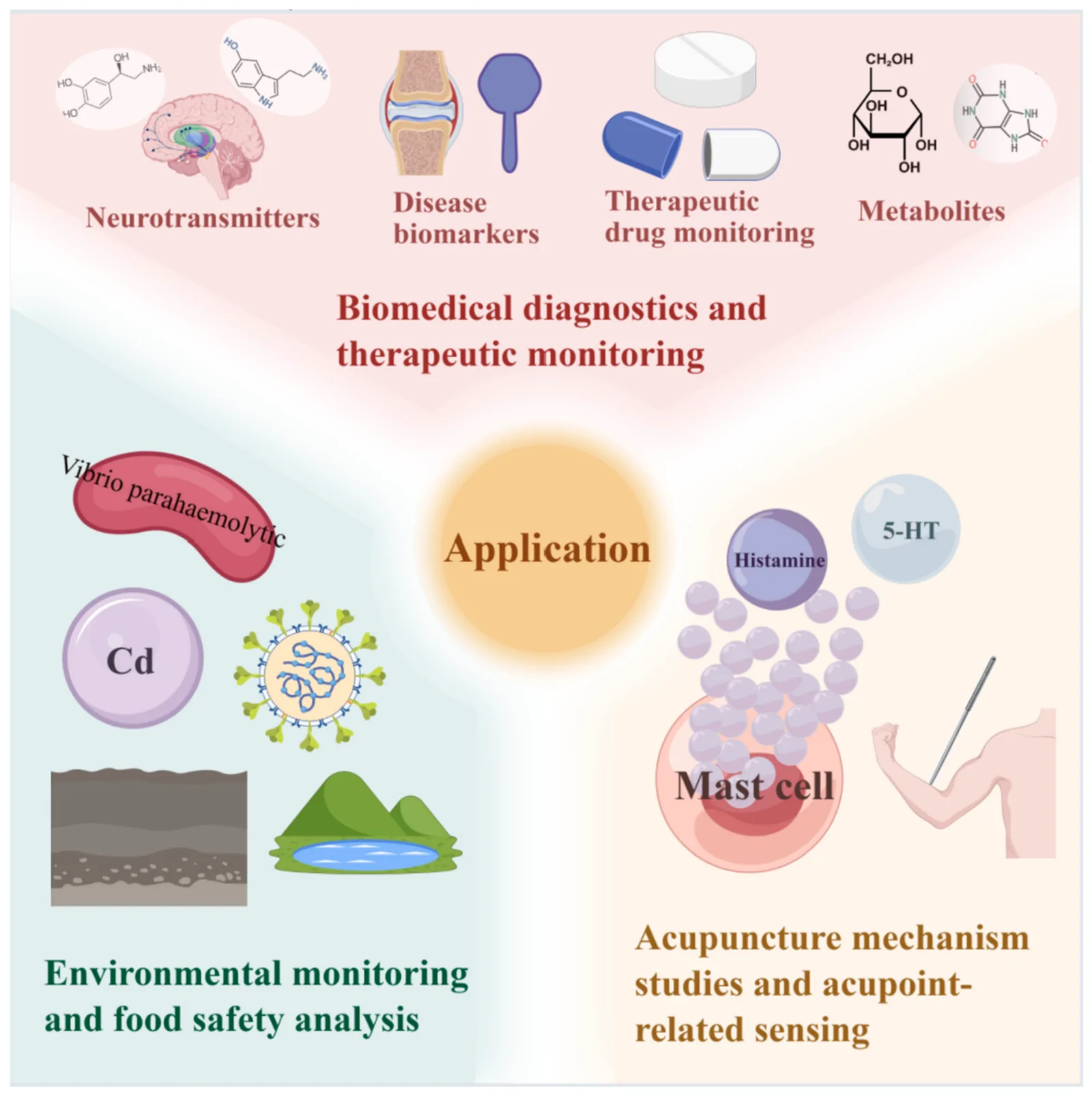
Applications of acupuncture needle-based biosensors.

The applications of ANBSs has expanded across diverse fields, including biomedical diagnostics, therapeutic monitoring, mechanistic studies of acupuncture, as well as environmental and food safety analysis. An overview of representative application areas of ANBSs, covering biomedical sensing, acupuncture mechanism investigation, and environmental/food safety analysis, is presented in Figure 4.

The applications discussed below were evaluated under different experimental conditions, including standard solutions, spiked biological samples, real biological specimens, living cells or ex vivo tissues, and animal in vivo studies. In this review, “real-time” refers to dynamic signal acquisition while the sensor remained in contact with a living biological system. For SERS-based ANBSs, studies in which the needle interacted with living tissue but Raman spectra were collected after needle withdrawal are described as “in vivo interaction followed by ex vivo readout”. Similarly, measurements using human serum, urine, or swab samples are considered ex vivo or sample-based validation unless the sensing was performed directly in a living human subject.

### 3.1. Biomedical Diagnostics and Therapeutic Monitoring

Among the currently reported application scenarios, biomedical analysis represented the most mature and intensively investigated direction for ANBSs. Benefiting from their needle-like geometry, minimally invasive insertion, and compatibility with in vivo operation, ANBSs have been employed for the detection of neurotransmitters, disease biomarkers, therapeutic drugs, and clinically relevant metabolites.

#### 3.1.1. Neurotransmitters

Neurotransmitters are endogenous chemical messengers that transmit, enhance, and convert specific signals between neurons and other cells. They included dopamine, acetylcholine, epinephrine, and norepinephrine. These molecules play important roles in many brain functions, including behavior and cognition. They also modulate muscle tone and heart rate, learning, sleeping, memory, consciousness, mood, and appetite [60]. Consequently, real-time monitoring of neurotransmitter dynamics is essential for understanding both physiological and pathological processes in the nervous system. ANBSs are well suited for this task due to their minimally invasive geometry and high spatial precision, which enable localized neurochemical measurements in biological tissues.

Among the neurotransmitters investigated, dopamine (DA) has been the most extensively studied. An representative example was reported by Tang et al., who fabricated a graphene-modified acupuncture needle for DA detection and successfully applied it to human serum analysis [4]. Subsequent studies had focused on improving sensitivity, selectivity, and in vivo applicability. For instance, Zhou et al. developed a field-effect transistor (FET)-based acupuncture needle neuroprobe that enabled highly sensitive DA detection and successfully differentiated dopamine release levels in the striatum between normal rats and Parkinson’s disease model rats [6]. Xu et al. constructed an MIP layer on MWCNT-functionalized acupuncture needles to achieve highly selective DA recognition [7]. In another study, Chen et al. integrated bimetallic nanoclusters with a molecularly imprinted interface to achieve the simultaneous detection of DA and chlorpromazine [3].

In addition to dopamine, serotonin (5-hydroxytryptamine, 5-HT) was another major target because of its central roles in mood regulation, sleep, appetite, and neurological disorders. A study by Li et al. developed a PEDOT/CNT-modified acupuncture needle microsensor for real-time in vivo monitoring of serotonin [8]. By using PEDOT as a conductive “glue” to stabilize CNTs on the needle tip, the authors obtained a mechanically robust interface that enabled direct monitoring of 5-HT at the Zusanli (ST36) acupoint in rats. More recent work had further improved analytical performance by combining Au/Pt bimetallic nanoparticles with a surface-imprinted recognition layer, enabling sensitive and selective 5-HT detection in serum, urine, and even PC12 cell systems [9].

Furthermore, monitoring of epinephrine (EP) and norepinephrine (NE) had also been reported. Zhan et al. fabricated a AuNP/PDA/AN microelectrode via gold nanoparticle electrodeposition and polydopamine electropolymerization, leveraging the synergistic catalytic effect for highly sensitive EP detection [46]. Zhou et al. developed a CNT/AuNT/AN sensor, modified with gold nanotubes and carbon nanotubes, to successfully monitor dynamic NE release in the locus coeruleus of rats in vivo [61]. These studies indicated that ANBSs are advancing toward higher sensitivity and selectivity, providing powerful tools for neuroscience research.

#### 3.1.2. Disease Biomarkers

Beyond neurotransmitters, ANBSs had also been extended to the detection of disease biomarkers, offering potential for early diagnosis and personalized medicine. For example, Kong et al. developed an osteopontin (OPN) sensor based on a surface molecularly-imprinted polymer (SMIP) layer and a built-in polymethylene blue probe on an acupuncture needle [19]. The sensor showed a linear response from 0.01 to 1000 ng/mL with a detection limit as low as 3 pg/mL and was successfully used for OPN determination in human serum. Zhang et al. also reported an acupuncture needle sensor for mesothelin (MSLN), a cancer-related biomarker, using Au nanoparticles and a functionalized polymer interface, achieving a detection limit of 10 pg mL^−1^ [17]. Moreover, ANBSs had demonstrated potential for detecting of biomarkers associated with neurodegenerative diseases. Kong et al. developed an electrochemical microbiosensor for β-amyloid_42_ (Aβ_42_) detection using an acupuncture needle microelectrode as the substrate [18]. In this design, AuNPs and SWCNTs were employed to construct a conductive interface, while a built-in hemin electroactive probe and an SMIP layer were integrated to achieve specific recognition and signal transduction toward Aβ_42_. The resulting sensor exhibited excellent sensitivity and selectivity, with a linear range from 100 to 1 × 10^10^ fM and a detection limit as low as 0.05 fM, and it was successfully applied to the analysis of real spiked samples. Although the number of reported examples remained limited, these studies indicated that ANBSs could be adapted for clinically relevant disease biomarker analysis through appropriate interfacial recognition design.

#### 3.1.3. Therapeutic Drug Monitoring

Therapeutic drug monitoring is another important application of ANBSs, particularly for drugs that require real-time concentration tracking to optimize efficacy and reduce side effects [62]. In this field, the structural advantages of ANBSs became evident, as they allowed direct access to biological compartments while maintaining relatively low invasiveness.

Monitoring of anticancer and neuropsychiatric drugs is crucial for personalized medicine. Liu et al. designed a “needle-in-needle” electrochemical sensor for in vivo monitoring of the anticancer drug etoposide [22]. A molecularly imprinted polymer (MIP) and Au nanoparticle-modified acupuncture needle (MIP@Au-CCP/AN) was housed inside a syringe needle. The outer syringe protected the delicate sensing layer during skin puncture, enabling long-term, stable monitoring of drug concentration in the peritoneal cavity of living rats. For psychotropic drugs, Liu and co-workers developed a chlorpromazine (CPZ) sensor based on an acupuncture needle electrode modified with Au/Cu bimetallic nanoparticles and a molecularly imprinted polymer (MIP) interface [3]. Benefiting from the synergistic electrocatalysis of the Au/Cu bimetal and the selective molecular recognition of the imprinted cavities, the sensor exhibited two linear ranges of 0.1–100 μM and 100–1000 μM, with a detection limit of 0.07 μM, and was successfully applied to human serum analysis. A similar strategy was reported for promethazine (PMZ) detection. Liu et al. fabricated an MIP/Au/Sn/ANE sensor by electrodepositing Au/Sn bimetallic nanoclusters on acupuncture needle, followed by electropolymerization of a surface-imprinted film [21]. The sensor showed a wide linear range of 0.5–500 μM and a detection limit of 0.14 μM, and it was successfully validated in human serum and environmental water.

#### 3.1.4. Metabolites

Among the metabolites investigated by ANBSs, glucose was the most extensively studied target. An example is the Pt/Au/AN non-enzymatic glucose sensor, in which Au nanoparticles and Pt nanoparticles were sequentially electrodeposited onto a stainless-steel acupuncture needle [16]. The resulting bimetallic interface served as an efficient electrocatalytic site for glucose oxidation and enabled glucose determination in human serum under near-physiological conditions (pH 7.4), with good selectivity and stability. This work highlighted the value of constructing noble-metal bimetallic interfaces on ANs for non-enzymatic glucose sensing.

Other bimetallic or mixed-metal architectures had further enhanced the performance of AN-based glucose sensors. For example, Chen et al. reported a pinecone-shaped Ni/Au/AN electrode in which Au/Ni nanostructures provided abundant catalytic sites and a synergistic electrocatalytic effect, resulting in a low detection limit of 0.14 μM and successful application in human serum [14]. More recently, Zhu et al. developed a CoCu-LDH/Cu_2_O/ANE platform featuring porous, ordered nanosheet structures that accelerated electron transfer and increased the number of active sites for glucose oxidation [15]. This sensor showed fast response and successful glucose determination in serum. All of these studies indicated that rationally designed multimetallic or mixed-valence nanostructures are highly effective for improving glucose sensing on acupuncture needle electrodes.

In addition to glucose, other clinically or analytically relevant metabolites have also been detected by ANBSs. Uric acid (UA) sensing has benefited from molecular imprinting strategies: Li et al. constructed an alumina-based inorganic MIP layer on an MWCNT-functionalized acupuncture needle microelectrode, enabling selective UA recognition with a linear range of 0.5–500 μM and a detection limit of 0.11 μM in serum and urine [43]. Ascorbic acid was another representative small biomolecule successfully detected using acupuncture-needle electrodes. Jia et al. fabricated an Ni_6_MnO_8_ nanoflake-modified acupuncture needle electrode that showed high electrocatalytic activity toward AA, with a sensitivity of 3106 μA·mM^−1^·cm^−2^ and a detection limit of 0.1 μM, and successfully applied it to vitamin C tablet analysis [58].

### 3.2. Acupuncture Mechanism Studies and Acupoint-Related Sensing

A particularly distinctive application of ANBSs lies in their use as analytical tools to elucidate the biological mechanisms of acupuncture itself. Unlike conventional biosensors, ANBSs could be directly inserted into acupoints or specific brain regions, enabling real-time and in vivo monitoring of biochemical signals triggered by acupuncture stimulation. Current studies in this area mainly cover two levels of investigation: monitoring biochemical responses in the local acupoint microenvironment and tracking neurochemical changes in acupuncture-related brain regions.

Among the local biochemical signals, serotonin (5-HT) and histamine are particularly representative because both are closely related to mast-cell degranulation induced by acupuncture manipulation. Li et al. reported an early and important example by developing a PEDOT/CNT-modified acupuncture needle for real-time in vivo monitoring of serotonin [8]. Their study emphasized that acupoint tissues were enriched with mast cells and that needling manipulation could promote mast-cell degranulation, thereby causing dynamic release of bioactive mediators such as 5-HT. Using this modified needle, they achieved direct real-time monitoring of serotonin in the Zusanli (ST36) acupoint of rats. The sensor exhibited detection limits of about 50 nM in PBS and 78 nM in cell medium, together with good selectivity against common inflammatory mediators and electroactive interferents. This work provided one of the earliest demonstrations that ANBSs could capture local molecular events at acupoints during needling.

More recently, Xu and co-workers further advanced local acupoint sensing by achieving the real-time in vivo monitoring of histamine at an acupoint using a graphene-modified acupuncture needle-based biosensor [28]. In this study, gold particles and graphene were sequentially introduced onto the needle surface to construct an AN/AuPs/G sensing platform with a detection limit of approximately 4.352 × 10^−12^ mol L^−1^ and a sensitivity of about 6.296 μA μM^−1^, while maintaining satisfactory specificity, repeatability, and stability. And, the authors compared the dynamic histamine responses between acupoint PC6 and a non-acupoint in rats. They observed only minimal changes at the non-acupoint, whereas histamine at PC6 showed a characteristic increase-then-decrease profile during acupuncture, with the peak appearing at around 18 min. This study pioneered the real-time monitoring of histamine at acupoints with high sensitivity and offered invaluable insights into the acupoint-activation mechanisms underlying acupuncture efficacy.

In addition to local acupoint sensing, ANBSs have also been applied to neurochemical monitoring in specific brain regions related to acupuncture efficacy. An example was reported by Pei et al., who developed an Ag_2_S/MoS_2_/GAN microneedle sensor for real-time monitoring of hydrogen sulfide (H_2_S) in the paraventricular nucleus (PVN) of hypertensive rats during electroacupuncture [2]. By sequentially integrating Au nanoparticles, MoS_2_, and Ag_2_S onto the needle surface, the authors constructed a platform capable of sensitive and selective H_2_S detection with good stability. Using this sensor, they found that electroacupuncture at ST36 (Zusanli) and LR3 (Taichong) significantly increased H_2_S levels in the PVN, and this neurochemical change was accompanied by a decrease in blood pressure. Combined with histological staining and Western blot analysis, the study further suggested that the antihypertensive effect of acupuncture might be associated with H_2_S-related signaling in the PVN. This work demonstrated that such sensors could directly track the molecular correlates of acupuncture-induced therapeutic effects in vivo.

Overall, current studies indicate that ANBSs provide a unique methodological advantage for acupuncture mechanism research because they allow real-time monitoring of biochemical responses at both the acupoint region and the central nervous system level. Local studies on serotonin and histamine mainly illuminate the acupoint activation mechanism accompanied by rapid release of mast-cell-related mediators, while central monitoring of H_2_S in the PVN helped explain how peripheral acupuncture stimulation regulated systemic physiological response, such as blood pressure reduction. Therefore, acupuncture needle-based biosensors are potential tools for decoding the biological basis of acupuncture.

### 3.3. Environmental Monitoring and Food Safety Analysis

Beyond biomedical applications, ANBSs have also shown considerable promise in environmental monitoring and food safety analysis. Their miniaturized geometry and adaptability to electrochemical or SERS-based detection make them suitable for the rapid analysis of heavy metal ions, pathogens, and organic contaminants in complex matrices.

#### 3.3.1. Heavy Metal Ions and Pathogens

Heavy metal ions are important environmental and foodborne pollutants because they are toxic, could accumulate along the food chain, and can ultimately threatened human health [63]. ANBSs have proven to be effective for heavy metal ion sensing. For example, Guo et al. developed a CS/COF/PEDOT:PSS-modified acupuncture needle electrode for Cd(II) detection in aquatic products [25]. In this design, PEDOT:PSS served as a conductive film to enhance charge transfer, whereas the CS/COFs layer increased the specific surface area. Using differential pulse anodic stripping voltammetry, the sensor showed a linear response from 400 to 2400 μg/L and a low detection limit of 13.8 ng/L, and it was successfully applied to scallop samples.

More recently, Guo et al. further extended this concept by constructing a self-calibrating ratiometric aptasensor on a needle electrode platform for the simultaneous detection of Cd(II) and Vibrio parahaemolyticus in aquatic products [26]. In this system, Zn-Co-MOF@AQ functioned not only as a signal-amplifying nanointerface but also as an internal reference because the embedded anthraquinone generated a stable reference signal. Meanwhile, methylene blue and tetramethylbenzidine were separately linked to aptamers targeting Cd(II) and Vibrio parahaemolyticus. By calculating the ratios of target-derived currents to the AQ reference current, the sensor achieved simultaneous detection with detection limits of 0.48 ng/L for Cd(II) and 1.39 CFU/mL for V. parahaemolyticus. This ratiometric self-calibration design could suppress background interference from the electrode material and environmental fluctuations to improve accuracy and reliability.

The detection of viral antigens further illustrated the applicability of ANBSs in complex biological samples. Chen et al. designed a dual-functional acupuncture needle biosensor for SARS-CoV-2 antigen detection by co-immobilizing antifouling polyethylene glycol (PEG) and SARS-CoV-2 antibodies on a poly(EDOT-COOH-co-EDOT-N_3_)-functionalized needle surface [40]. This sensor combined ultrasensitive detection with strong antifouling capability, thereby overcoming one of the major limitations of electrochemical biosensors in real samples: nonspecific adsorption. This study suggested that future development of acupuncture needle-based pathogen sensors should focus not only on sensitivity enhancement but also increasingly on interface antifouling and analytical reliability in complex matrices.

#### 3.3.2. Organic Pollutants

Beyond heavy metals, ANBSs, particularly those leveraging SERS, are powerful tools for detecting a wide array of organic chemical contaminants. A SERS-active acupuncture silver needle, carved with micron-sized grooves to trap Au nanoparticles, was developed for the detection of malachite green, a prohibited carcinogen in aquaculture [50]. The microgrooves protected the AuNPs from being wiped off during insertion, enabling direct insertion into fish tissue for label-free detection with a detection limit as low as 0.1 nM.

In addition to SERS-based strategies, molecular imprinting has emerged as another approach for extending ANBSs to small-molecule organic contaminants. Liu et al. fabricated a highly selective trimethoprim (TMP) sensor based on an acupuncture needle microelectrode functionalized with three-dimensional coral-like Au nanorods, polydopamine, and an electropolymerized molecularly imprinted polypyrrole layer [24]. The key design principle was the formation of surface nanocavities complementary to the structure of TMP, which conferred both molecular selectivity and favorable electron-transfer characteristics. The sensor showed a wide linear range of 0.05–50 μmol L^−1^ and a detection limit of 0.017 μmol L^−1^, and it was successfully applied to lake water, soil, and pharmaceutical capsule samples.

Applications of ANBSs have expanded to biomedical diagnosis, therapeutic monitoring, mechanistic acupuncture research, and environmental/food safety analysis. Among these fields, biomedical sensing and acupuncture mechanism studies are the most distinctive and representative because they exploit the inherent advantages of the needle-shaped platform, including minimal invasiveness, spatial precision, and compatibility with in vivo operation. At the same time, applications in food and environmental analysis revealed the versatility of AN-based interfaces in dealing with complex matrices. Future development in this field would likely move toward multiplexed sensing, antifouling platforms, long-term in vivo monitoring, and portability.

## 4. Challenges and Future Directions of ANBSs

Despite considerable advances, ANBSs still face several substantial challenges prior to broader practical or clinical implementation. First, the mechanical robustness of the modified layer on acupuncture needle remains a major bottleneck. To improve sensitivity, nanomaterials are often coated onto or electrodeposited on the needle surface. However, during skin penetration or repeated insertion, these surface coatings might crack, delaminate, or peel off, leading to signal fluctuation, loss of active materials, and even potential biosafety concerns due to residual nanomaterials left in tissue [22].

Second, long-term stability in vivo is severely limited by biofouling and interfacial degradation. Once exposed to real biological environments, the sensor surface readily adsorbs proteins, cells, lipids, and extracellular components, which gradually form an insulating layer [30,64,65]. This biofouling process passivates the interface, increases charge-transfer resistance, and causes signal attenuation or drift. These issues are often underestimated in in vitro studies conducted in idealized buffer systems or artificial samples, meaning that laboratory performance might not directly translate to real physiological settings [31].

Third, the long-term reliability of biorecognition elements remains problematic. When enzymes are used as recognition units, their denaturation, deactivation, or gradual leaching could reduce both sensitivity and specificity over time [66]. Although aptamers and molecularly imprinted polymers (MIPs) offer alternatives with better structural tunability or higher thermal stability, they also require careful interface design, immobilization control, and optimization of signal transduction efficiency [31]. Thus, preserving recognition performance while maintaining long-term robustness remained a key material and interface challenge.

Finally, integration and portability are still insufficient. Many reported ANBSs still rely on a conventional three-electrode system, in which both the working and reference electrodes must be implanted or positioned separately, increasing invasiveness and operational complexity [11]. Moreover, ideal clinical testing devices should be simple, convenient, and easy to operate for rapid on-site testing, yet some existing electrochemical devices still require support from a laboratory environment. Although electrochemical sensors theoretically offer advantages of speed and sensitivity, their complex preparation and detection steps might limit their diffusion in practical clinical applications [67].

Looking forward, the future development of ANBSs might proceed along several interconnected directions. One important route is the development of advanced functional materials with improved conductivity, flexibility, biocompatibility, and structural robustness, such as two-dimensional nanomaterials, porous conductive frameworks, degradable polymers, liquid metals, and hybrid nanocomposites [30,68]. Rational material combinations might help overcome the limitations of single-component systems and improve the overall balance among sensitivity, selectivity, stability, mechanical durability, and multifunctional sensing.

Another important direction is surface/interface engineering. Introducing hydrophilic groups, antifouling coatings, porous microstructures, or roughened electrode architectures might improve wettability, reduce nonspecific adsorption, and stabilize signal transduction in complex media. Likewise, better immobilization strategies for enzymes, aptamers, antibodies, or MIPs are needed to preserve recognition performance without sacrificing interfacial conductivity [31].

A further important direction involves computational and intelligent sensing strategies. Machine learning and chemometric methods had been proposed for drift correction, adaptive calibration, and feature extraction in continuous electrochemical monitoring [62,64].

## 5. Conclusions

Overall, acupuncture needle-based biosensors have been developed from simple modified needle electrodes into multifunctional sensing platforms that combine functional materials, recognition elements, and different signal transduction strategies. The small size, metallic conductivity, mechanical strength, ease of surface modification, and ability of acupuncture needles to access biological tissues make them attractive substrates for minimally invasive sensing. Current studies have demonstrated their potential in the detection of neurotransmitters, disease biomarkers, therapeutic drugs, metabolites, environmental contaminants, and food-related targets, as well as in the investigation of biochemical changes associated with acupuncture. Electrochemical sensing remains the most widely used strategy because of its relatively simple instrumentation and quantitative readout, while SERS-based sensing provides complementary advantages in highly sensitive and molecularly specific detection.

Despite these advances, several limitations still restrict the further development of ANBSs. The stability of functional coatings during tissue insertion, biofouling, signal drift, long-term sensing stability, and reproducibility remain important challenges. In addition, many reported systems still require further validation in complex biological environments and in vivo conditions. Therefore, future research should focus on improving functional materials and interface stability, and enhancing antifouling performance. With continued advances in materials and sensing interfaces, ANBSs might become useful tools for minimally invasive biochemical monitoring and for studying acupuncture-related physiological processes. By summarizing current sensing strategies, representative applications, and remaining challenges, this review provides a practical reference for the further design and development of ANBSs.

## Figures and Tables

**Table 1:** Classification of needle-based sensing platforms discussed in this review.

Device Category	Operational Definition in This Review	Role of the Acupuncture Needle	Included as ANBS	Representative Configuration
Conventional acupuncture needle sensor	A commercial or conventional acupuncture needle was directly functionalized and used as the principal sensing substrate	Working electrode or optical/SERS probe carrier	Yes	Functionalized acupuncture needle electrode [4,13]
Acupuncture needle-based “microneedle”	The study described the device as a microneedle, but the sensing part was based on an acupuncture needle	Sensing substrate	Yes	Functionalized acupuncture needle used for pH [10,11]
Integrated ANBS	An acupuncture needle was combined with other components for protection, signal measurement, or device integration	Sensing substrate	Yes	Dual-needle FET [12], needle-in-needle [22], capillary-integrated sensor [36]
Syringe/hypodermic needle sensor	A syringe or hypodermic needle was used as the sensing substrate without an acupuncture needle	No acupuncture needle involved in sensing	No	Syringe-needle electrochemical sensor
Wire microelectrode	A metallic or carbon wire served as the primary sensing electrode without an acupuncture needle	No acupuncture needle involved in sensing	No	Metal-wire or carbon-fiber microelectrode
Microfabricated microneedle patch/array	Microscale needles or arrays were separately fabricated for sampling or sensing	No acupuncture needle involved in sensing	No	porous, hydrogel, or coated microneedle array

**Table 2:** Representative analytical performance of electrochemical ANBSs.

Study	Sensor Design	Target Analyte	Sample/Test Condition	Linear Range	LOD	Performance Evaluation	Validation Level
Li et al., 2016 [8]	PEDOT/CNT-functionalized AN	5-HT	PBS, cell medium; rat acupoint	NR	50 nM in PBS; 78 nM in cell medium	Selectivity and stability evaluated	Animal in vivo, real-time
Tang et al., 2017 [59]	FGPC/Au-functionalized AN	NO	PBS; rat acupoints	NR	3.2 nM	Selectivity evaluated	Animal in vivo, real-time
Zhou et al., 2018 [10]	PAN/MoS_2_/AN	pH	PBS; rat brain	pH 3.0–9.0	Not applicable for pH	Stable response reported	Animal in vivo, real-time
Liu et al., 2024 [22]	MIP@Au-CCP AN inside syringe needle	Etoposide	Buffer, peritoneal fluid; living rats	0.1–160 μM in buffer; 17–3401 μM in undiluted peritoneal fluid	0.05 μM in buffer	Repeatability RSD 3.7%; reproducibility RSD 4.1%; 88.3% signal retained after 16 days	Animal in vivo
Zhan et al., 2024 [9]	Au/Pt + MIP functionalized AN	5-HT	Human serum, urine; stimulated PC12 cells	0.03–500 μM	0.0106 μM	Selectivity, stability and reproducibility reported	Real biofluid + living cells; not animal in vivo
Chen et al., 2025 [40]	AuNP/poly(EDOT)-antibody-PEG AN	SARS-CoV-2 antigen	Swab samples	1 ag/mL^−1^–1 ng/mL^−1^	0.21 ag/mL^−1^	RSD < 4.2% in swabs	Real clinical-type specimen, not in vivo
Pei et al., 2025 [2]	AuNP/MoS_2_/Ag_2_S AN	H_2_S	Rat PVN	1–100 μM	95 nM	Effective for up to 2 weeks at room temperature	Animal in vivo, real-time
Kong et al., 2026 [18]	ANME/AuNPs/SWCNTs~hemin~Aβ42@SMIP	Aβ42	Real spiked samples	100–10^10^ fM	0.05 fM	Selectivity, stability and reproducibility reported	Spiked sample; not in vivo
Kong et al., 2025 [19]	SMIP + built-in pMB probe AN	Osteopontin	Analytical samples	0.01–1000 ng/mL^−1^	3 pg/mL^−1^	Selectivity and stability reported	In vitro

Note: AN, acupuncture needle; LOD, limit of detection; NR, not reported; RSD, relative standard deviation; MIP, molecularly imprinted polymer. Linear ranges and LODs are reported in the original units used in the cited studies. “Animal in vivo, real-time” indicates that signals were recorded while the sensor was placed in a living animal. Studies using serum, urine, swab samples, or other collected biological specimens are not classified as in vivo sensing.

**Table 3:** Representative analytical performance of SERS-based ANBSs.

Study	Sensor Design	Target Analyte	Sample/Test Condition	Linear Range	LOD	Performance Evaluation	Validation Level
Li et al., 2017 [48]	AuNP/CA-Fe(III)-based SERRS	Dopamine	Pretreated serum and mouse CSF	NR	0.1 nM in serum; 2.5 nM in CSF	Selective recognition and reproducible SERRS response reported	Ex vivo biological samples
Zhou et al., 2019 [50]	Au NPs-groove-trapped Ag needles (Au-G-AgNs)	Malachite green	Fish tissue	NR	0.1 nM	PVP used as an internal reference; RSD < 10% for 30 spectra	In vivo sampling/ex vivo readout
Chen et al., 2021 [52]	Magnetic SERS probes embedded in a needle pit	pH, redox potential, NO	Living tissue	NR	NR	Magnetic pit improved probe retention during tissue insertion	In vivo interaction/ex vivo readout
Xiong et al., 2025 [53]	Dual-groove gold nanoshell SERS probes	H_2_O_2_ and redox potential	Rabbit wounds	NR	NR	Groove-confined probes improved retention during tissue insertion	In vivo interaction/ex vivo readout
Zhong et al., 2026 [55]	ANs@Au NPs@MNBA	pH + NTR	Solution and intact tissue	pH 4.75–7.25; NTR 0–20 μg/mL	NR	Au-S signal used as an internal reference for ratiometric analysis	In vitro + ex vivo tissue

Note: AN, acupuncture needle; SERS, surface-enhanced Raman spectroscopy; SERRS, surface-enhanced resonance Raman spectroscopy; LOD, limit of detection; NR, not reported; RSD, relative standard deviation; NTR, nitroreductase. “In vivo interaction/ex vivo readout” indicates that the needle interacted with the target environment in a living animal, while Raman spectra were collected after needle removal. Such studies are not considered continuous real-time in vivo Raman monitoring.

## Data Availability

No new data were created or analyzed in this study. Data sharing is not applicable to this article.
